# Supplementary material for: Functional genomics of lipid metabolism in the oleaginous yeast Rhodosporidium toruloides
Source: eLife. 2018 Mar 9;7:e32110. doi: 10.7554/eLife.32110 (PMC5922974; doi:10.7554/eLife.32110)
Supplement: Transparent reporting form [file elife-32110-transrepform.pdf]

eLIFE
1st Floor
24 Hills Road
Cambridge CB2 1JP, UK
P 01223 855340
W elifesciences.org
T @elife

eLife's transparent reporting form

We encourage authors to provide detailed information *within their submission* to facilitate the interpretation and replication of experiments. Authors can upload supporting documentation to indicate the use of appropriate reporting guidelines for health-related research (see EQUATOR Network), life science research (see the BioSharing Information Resource), or the ARRIVE guidelines for reporting work involving animal research. Where applicable, authors should refer to any relevant reporting standards documents in this form.

If you have any questions, please consult our Journal Policies and/or contact us: editorial@elifesciences.org.

Sample-size estimation

- You should state whether an appropriate sample size was computed when the study was being designed
- You should state the statistical method of sample size computation and any required assumptions
- If no explicit power analysis was used, you should describe how you decided what sample (replicate) size (number) to use

Please outline where this information can be found within the submission (e.g., sections or figure legends), or explain why this information doesn't apply to your submission:

This study consist primarily of three types of data: Relative strain abundance in mixed cultures as measured by sequencing unique strain barcodes, relative growth between pure cultures as measured by optical density, and relative lipid content as measured by relative intensity of neutral lipid-staining fluorescent dye.

Relative strain abundance

This study introduces a new technique to construct barcoded pools of mutant strains in a non-model fungus to enable high-throughput functional profiling of gene function. As such, one of our primary objectives was to establish experimental strategies and statistical thresholds to detect functionally important genes with high confidence, while keeping experimental logistics to a minimum to enable the widest application of this technology.

A theoretical power analysis would have been highly uncertain due to the many possible types of confounding mutations in the mutant pool. These mutations are discussed in Appendix 1, lines 1804-1826, where we explain that our goal was to map as many T-DNA insertions per gene as was practical to mitigate any confounding phenotypes. We report the distribution of independent T-DNA inserts that were tracked per gene in Appendix 1 (lines 1858-1866) and report the number inserts tracked for every gene with BarSeq data in Supplementary file 2. As we state on line 356 in the main text:
"To establish if RB-TDNAseq could produce statistically robust results with minimal experimental replication, we recovered three independent starter cultures from frozen aliquots of the mutant pool and used each replicate to inoculate both supplemented and non-supplemented cultures."

Relative growth measurements

We used optical density as measure of growth rate on fatty acids to validate our fitness results. For these experiments we expected relatively large differences in strain fitness and saw no need for any formal power analysis.

Relative lipid measurements

For each targeted deletion mutant, we compared BODIPY signal between the mutant strain and parental *YKU70Δ* strain. Based on observations from pilot experiments, we chose to test six biological replicates for each mutant, processed on at least two different days. This choice was made with only limited data on the variation to be expected in this data and thus an a priori power analysis was not feasible. A post-hoc power analysis of this sample strategy is described in Appendix 1 (line 2105)

Replicates

- You should report how often each experiment was performed
- You should include a definition of biological versus technical replication

- The data obtained should be provided and sufficient information should be provided to indicate the number of independent biological and/or technical replicates
- If you encountered any outliers, you should describe how these were handled
- Criteria for exclusion/inclusion of data should be clearly stated
- High-throughput sequence data should be uploaded before submission, with a private link for reviewers provided (these are available from both GEO and ArrayExpress)

Please outline where this information can be found within the submission (e.g., sections or figure legends), or explain why this information doesn't apply to your submission:

Replication is explicitly shown in all charts and figures.

We define our use of the term 'biological replicate' in the Methods in the subsection 'Culture conditions'

Outliers were not removed from any of our datasets. The BarSeq analysis algorithms we adapted from Wetmore et al (2015) are designed to reduce the influence of outlier insertion strains on fitness estimates, however. Those algorithms are described in detail in the referenced publications.

No data were excluded from the experiments presented, with the exception of three sets of cultures excluded from BODIPY measurements on lipid accumulation mutants due to contamination. These are documented in the raw BODIPY data reported in Supplementary file 2.

High throughput sequencing data used for improved genome assembly, TnSeq and BarSeq are available on the NCBI Short Read Archive with relevant accession numbers given for each sequence type in the Methods

Statistical reporting

- Statistical analysis methods should be described and justified
- Raw data should be presented in figures whenever informative to do so (typically when N per group is less than 10)
- For each experiment, you should identify the statistical tests used, exact values of N, definitions of center, methods of multiple test correction, and dispersion and precision measures (e.g., mean, median, SD, SEM, confidence intervals; and, for the major substantive results, a measure of effect size (e.g., Pearson's r, Cohen's d)
- Report exact p-values wherever possible alongside the summary statistics and 95% confidence intervals. These should be reported for all key questions and not only when the p-value is less than 0.05.

Please outline where this information can be found within the submission (e.g., sections or figure legends), or explain why this information doesn't apply to your submission:

We used a moderated T-statistic in our analysis of BarSeq data, consistent with previous work in bacteria. These statistics are described in detail in Wetmore et al (2015) and briefly reviewed in the Materials and Methods. We explicitly demonstrate the utility of those statistics in the Results subsection "Mapping biosynthetic pathways using RB-TDNAseq". T-statistics are reported for all BarSeq experiments in Supplementary file 2.

Heat maps of BarSeq data show fitness scores averaged across all T-DNA inserts for each biological replicates, as showing data from each insert is impractical and would make interpretation of figures very difficult. Readers interested in the insert-level data can access it at the interactive fitness browser (http://fungalfit.genomics.lbl.gov/). Measurements from individual biological replicates are explicitly shown in figures of optical density and BODIPY data. N, P values and effect size (Cohen's D) are for all strains and time points in Figure 5B, Figure 5C, Figure 3B, Figure 3C, and Figure 3 – figure supplement 3 are reported in Supplementary file 2, referenced in the appropriate figure legends.

(For large datasets, or papers with a very large number of statistical tests, you may upload a single table file with tests, Ns, etc., with reference to sections in the manuscript.)

Group allocation

- Indicate how samples were allocated into experimental groups (in the case of clinical studies, please specify allocation to treatment method); if randomization was used, please also state if restricted randomization was applied
- Indicate if masking was used during group allocation, data collection and/or data analysis

Please outline where this information can be found within the submission (e.g., sections or figure legends), or explain why this information doesn't apply to your submission:

For the most part, samples in this study were not allocated into experimental groups. BODIPY measurements shown in Figure 5 were gathered in batches, however, due to logistical constraints. Strains were not explicitly randomized, but each batch included strains with a mix of expected phenotypes. This data collection strategy is discussed in the methods section.

No masking was used in this study.

eLife Sciences Publications, Ltd is a limited liability non-profit non-stock corporation incorporated in the State of Delaware, USA, with company number 5030732, and is registered in the UK with company number FC030576 and branch number BR015634 at the address 1st Floor, 24 Hills Road, Cambridge CB2 1JP | August 2014

Additional data files ("source data")

- We encourage you to upload relevant additional data files, such as numerical data that are represented as a graph in a figure, or as a summary table
- Where provided, these should be in the most useful format, and they can be uploaded as "Source data" files linked to a main figure or table
- Include model definition files including the full list of parameters used
- Include code used for data analysis (e.g., R, MatLab)
- Avoid stating that data files are "available upon request"

Please indicate the figures or tables for which source data files have been provided:

In addition to the source data included in Supplementary files 1 – 6 included in this submission, we have uploaded raw data and code used for analysis at the following online repositories:

https://www.ncbi.nlm.nih.gov/sra
NCBI Short Read Archive (SRA):
Genome sequence: SRP114401 and SRP058059
TnSeq data: SRP116146
BarSeq data: SRP116193

https://bitbucket.org/FungalTDNAseq/
Atlassian BitBucket Code Repository
Code and data used to identify fused gene models:
https://bitbucket.org/FungalTDNAseq/fusedgenemodels

Code used to map T-DNA insertions and analyze BarSeq data, as well as raw data and analysis scripts for BODIPY measurements:
https://bitbucket.org/FungalTDNAseq/rb-tdnaseq

Fitness scores and T-statistics for all genes and conditions, as well as strain-level abundance data:
http://fungalfit.genomics.lbl.gov/cgi-bin/org.cgi?orgId=Rtoru

eLife Sciences Publications, Ltd is a limited liability non-profit non-stock corporation incorporated in the State of Delaware, USA, with company number 5030732, and is registered in the UK with company number FC030576 and branch number BR015634 at the address 1st Floor, 24 Hills Road, Cambridge CB2 1JP | August 2014
